# Supplementary material for: The influence of management practices on plant diversity: a comparative study of three urban wetlands in an expanding city in eastern China
Source: PeerJ. 2024 Jan 4;12:e16701. doi: 10.7717/peerj.16701 (PMC10771763; doi:10.7717/peerj.16701)
Supplement: Supplemental Information 1 [file peerj-12-16701-s001.docx]

**Table S1：The habitat features of 81 sample plots in three urban wetlands**

| **Names** | **No** |  | **Geographic**  **information** | |  | **Natural**  **environmental factors** | | | | |  | **Artificial**  **interference factors** | | | |  | **Information of diversity index and coverage** | | | |
| --- | --- | --- | --- | --- | --- | --- | --- | --- | --- | --- | --- | --- | --- | --- | --- | --- | --- | --- | --- | --- |
|  |  |  | **Long** | **Lat** |  | **Pre** | **AAT** | **Alt** | **WT** | **WSI** |  | **HA** | **CILUD** | **PSP** | **ICP** |  | **D** | **H** | **J** | **TC** |
| Xixi wetland | 1 |  | 120.0796 | 30.2783 |  | 1399 | 15.17 | 3 | 50 | 1.08 |  | 18 | 253.33 | 49 | 52 |  | 101 | 4.11 | 0.453 | 180 |
|  | 2 |  | 120.0818 | 30.2788 |  | 1399 | 15.17 | 4 | 47 | 1.21 |  | 18 | 293.71 | 50 | 34 |  | 84 | 4.03 | 0.429 | 120 |
|  | 3 |  | 120.0859 | 30.2801 |  | 1399 | 15.17 | 4 | 65 | 1.16 |  | 18 | 309.43 | 49 | 16 |  | 65 | 3.42 | 0.387 | 140 |
|  | 4 |  | 120.0899 | 30.2813 |  | 1399 | 15.17 | 5 | 70 | 1.61 |  | 18 | 207.11 | 67 | 13 |  | 80 | 3.75 | 0.399 | 98 |
|  | 5 |  | 120.0953 | 30.2786 |  | 1399 | 15.17 | 3 | 50 | 1.51 |  | 18 | 228.89 | 44 | 27 |  | 71 | 3.57 | 0.394 | 80 |
|  | 6 |  | 120.0924 | 30.2789 |  | 1399 | 15.17 | 4 | 82 | 1.79 |  | 18 | 211.56 | 51 | 11 |  | 62 | 3.33 | 0.381 | 55 |
|  | 7 |  | 120.0894 | 30.2796 |  | 1399 | 15.17 | 3 | 65 | 1.25 |  | 18 | 205.78 | 41 | 10 |  | 51 | 2.99 | 0.354 | 65 |
|  | 8 |  | 120.0863 | 30.2788 |  | 1399 | 15.17 | 3 | 60 | 1.32 |  | 18 | 232.00 | 28 | 23 |  | 51 | 2.99 | 0.354 | 45 |
|  | 9 |  | 120.0809 | 30.2751 |  | 1399 | 15.17 | 4 | 70 | 1.77 |  | 18 | 241.78 | 45 | 32 |  | 77 | 3.69 | 0.399 | 170 |
|  | 10 |  | 120.0829 | 30.2696 |  | 1399 | 15.17 | 4 | 55 | 1.32 |  | 18 | 216.67 | 38 | 15 |  | 53 | 3.05 | 0.360 | 150 |
|  | 11 |  | 120.0813 | 30.2705 |  | 1399 | 15.17 | 4 | 66 | 1.11 |  | 18 | 212.89 | 47 | 20 |  | 67 | 3.47 | 0.390 | 110 |
|  | 12 |  | 120.0786 | 30.2711 |  | 1399 | 15.17 | 5 | 70 | 1.14 |  | 18 | 215.33 | 37 | 10 |  | 47 | 2.84 | 0.342 | 70 |
|  | 13 |  | 120.0776 | 30.2741 |  | 1399 | 15.17 | 4 | 70 | 1.63 |  | 18 | 221.11 | 42 | 23 |  | 65 | 3.42 | 0.387 | 130 |
|  | 14 |  | 120.0782 | 30.2779 |  | 1399 | 15.17 | 4 | 59 | 1.18 |  | 18 | 234.89 | 50 | 13 |  | 63 | 3.36 | 0.383 | 110 |
|  | 15 |  | 120.0763 | 30.2822 |  | 1399 | 15.17 | 5 | 70 | 1.19 |  | 18 | 208.00 | 54 | 24 |  | 78 | 3.71 | 0.399 | 85 |
|  | 16 |  | 120.0743 | 30.2851 |  | 1399 | 15.17 | 4 | 50 | 1.36 |  | 18 | 231.11 | 33 | 7 |  | 40 | 2.56 | 0.315 | 75 |
|  | 17 |  | 120.0688 | 30.2853 |  | 1399 | 15.17 | 4 | 70 | 1.27 |  | 18 | 220.00 | 55 | 17 |  | 72 | 3.59 | 0.395 | 60 |
|  | 18 |  | 120.0657 | 30.2837 |  | 1399 | 15.17 | 1 | 40 | 1.17 |  | 18 | 221.78 | 1 | 1 |  | 2 | 0.60 | 0.091 | 40 |
|  | 19 |  | 120.0643 | 30.2807 |  | 1399 | 15.17 | 2 | 80 | 1.15 |  | 18 | 200.00 | 42 | 0 |  | 42 | 2.65 | 0.323 | 95 |
|  | 20 |  | 120.0571 | 30.2810 |  | 1399 | 15.17 | 2 | 75 | 1.35 |  | 18 | 235.56 | 43 | 19 |  | 62 | 3.33 | 0.381 | 140 |
|  | 21 |  | 120.0551 | 30.2667 |  | 1399 | 15.17 | 4 | 70 | 1.21 |  | 18 | 208.44 | 30 | 9 |  | 39 | 2.52 | 0.311 | 78 |
|  | 22 |  | 120.0536 | 30.2683 |  | 1399 | 15.17 | 2 | 55 | 1.11 |  | 18 | 200.00 | 30 | 25 |  | 55 | 3.25 | 0.422 | 75 |
|  | 23 |  | 120.0514 | 30.2665 |  | 1399 | 15.17 | 6 | 50 | 1.15 |  | 18 | 226.67 | 32 | 12 |  | 44 | 2.73 | 0.331 | 70 |
|  | 24 |  | 120.0517 | 30.2638 |  | 1399 | 15.17 | 2 | 70 | 1.11 |  | 18 | 200.00 | 42 | 24 |  | 66 | 3.44 | 0.388 | 70 |
|  | 25 |  | 120.0512 | 30.2572 |  | 1399 | 15.17 | 7 | 60 | 1.09 |  | 18 | 224.44 | 60 | 18 |  | 78 | 3.71 | 0.399 | 130 |
|  | 26 |  | 120.0543 | 30.2544 |  | 1399 | 15.17 | 6 | 55 | 1.20 |  | 18 | 208.44 | 49 | 18 |  | 67 | 3.31 | 0.386 | 80 |
|  | 27 |  | 120.0566 | 30.2548 |  | 1399 | 15.17 | 7 | 50 | 1.09 |  | 18 | 226.67 | 39 | 14 |  | 53 | 3.05 | 0.360 | 50 |
|  | 28 |  | 120.0592 | 30.2572 |  | 1399 | 15.17 | 7 | 65 | 1.11 |  | 18 | 210.00 | 30 | 16 |  | 46 | 2.80 | 0.338 | 40 |
|  | 29 |  | 120.052 | 30.2703 |  | 1399 | 15.17 | 2 | 75 | 1.12 |  | 18 | 217.78 | 34 | 14 |  | 48 | 2.88 | 0.345 | 78 |
|  | 30 |  | 120.0528 | 30.2747 |  | 1399 | 15.17 | 2 | 38 | 1.22 |  | 18 | 210.00 | 28 | 9 |  | 37 | 2.44 | 0.303 | 55 |
|  | 31 |  | 120.0683 | 30.2627 |  | 1399 | 15.17 | 2 | 50 | 1.18 |  | 18 | 382.22 | 27 | 12 |  | 39 | 2.52 | 0.311 | 80 |
|  | 32 |  | 120.0724 | 30.2641 |  | 1399 | 15.17 | 4 | 65 | 1.06 |  | 18 | 281.33 | 47 | 16 |  | 63 | 3.36 | 0.383 | 110 |
|  | 33 |  | 120.0673 | 30.2655 |  | 1399 | 15.17 | 7 | 75 | 1.08 |  | 18 | 227.33 | 30 | 17 |  | 47 | 2.84 | 0.342 | 90 |
|  | 34 |  | 120.0669 | 30.2667 |  | 1399 | 15.17 | 6 | 60 | 1.16 |  | 18 | 208.00 | 18 | 16 |  | 34 | 1.78 | 0.239 | 100 |
|  | 35 |  | 120.0665 | 30.2735 |  | 1399 | 15.17 | 3 | 79 | 1.15 |  | 18 | 213.11 | 54 | 19 |  | 73 | 3.61 | 0.396 | 110 |
|  | 36 |  | 120.0675 | 30.2761 |  | 1399 | 15.17 | 2 | 65 | 1.11 |  | 18 | 248.44 | 39 | 12 |  | 51 | 2.99 | 0.354 | 130 |
|  | 37 |  | 120.0697 | 30.2774 |  | 1399 | 15.17 | 4 | 55 | 1.80 |  | 18 | 277.78 | 29 | 12 |  | 41 | 3.19 | 0.377 | 120 |
|  | 38 |  | 120.0737 | 30.278 |  | 1399 | 15.17 | 6 | 77 | 1.10 |  | 18 | 225.33 | 38 | 15 |  | 53 | 3.05 | 0.360 | 130 |
|  | 39 |  | 120.076 | 30.2746 |  | 1399 | 15.17 | 4 | 55 | 1.14 |  | 18 | 214.00 | 46 | 10 |  | 56 | 3.15 | 0.368 | 95 |
|  | 40 |  | 120.0755 | 30.267 |  | 1399 | 15.17 | 4 | 82 | 1.15 |  | 18 | 248.89 | 28 | 9 |  | 37 | 2.44 | 0.303 | 80 |
|  |  |  |  |  |  |  |  |  |  |  |  |  |  |  |  |  |  |  |  |  |
| Tongjian Lake wetland | 1 |  | 120.0319 | 30.0718 |  | 1454 | 18 | 7 | 38 | 1.11 |  | 6 | 219.56 | 8 | 4 |  | 12 | 1.20 | 0.169 | 90 |
|  | 2 |  | 120.0334 | 30.0756 |  | 1454 | 18 | 14 | 55 | 1.15 |  | 6 | 231.11 | 22 | 32 |  | 54 | 2.67 | 0.330 | 83 |
|  | 3 |  | 120.0335 | 30.0754 |  | 1454 | 18 | 12 | 35 | 1.26 |  | 6 | 225.11 | 26 | 20 |  | 46 | 2.60 | 0.313 | 78 |
|  | 4 |  | 120.0335 | 30.0752 |  | 1454 | 18 | 11 | 45 | 1.39 |  | 6 | 227.56 | 27 | 34 |  | 61 | 2.77 | 0.339 | 159 |
|  | 5 |  | 120.0338 | 30.0748 |  | 1454 | 18 | 9 | 45 | 1.44 |  | 6 | 210.00 | 30 | 19 |  | 49 | 2.64 | 0.320 | 65 |
|  | 6 |  | 120.0339 | 30.0745 |  | 1454 | 18 | 8 | 40 | 1.53 |  | 6 | 240.89 | 23 | 24 |  | 47 | 2.66 | 0.313 | 140 |
|  | 7 |  | 120.0341 | 30.0745 |  | 1454 | 18 | 8 | 48 | 1.68 |  | 6 | 208.89 | 24 | 25 |  | 49 | 2.64 | 0.320 | 68 |
|  | 8 |  | 120.0346 | 30.0746 |  | 1454 | 18 | 10 | 55 | 1.40 |  | 6 | 239.56 | 26 | 22 |  | 48 | 2.63 | 0.318 | 78 |
|  | 9 |  | 120.0348 | 30.0743 |  | 1454 | 18 | 8 | 30 | 1.18 |  | 6 | 234.44 | 17 | 13 |  | 30 | 2.17 | 0.267 | 75 |
|  | 10 |  | 120.0348 | 30.0740 |  | 1454 | 18 | 7 | 35 | 1.19 |  | 6 | 222.67 | 19 | 24 |  | 43 | 2.55 | 0.306 | 83 |
|  | 11 |  | 120.0351 | 30.0731 |  | 1454 | 18 | 11 | 30 | 1.42 |  | 6 | 230.00 | 26 | 19 |  | 45 | 2.59 | 0.311 | 105 |
|  | 12 |  | 120.0355 | 30.0726 |  | 1454 | 18 | 7 | 28 | 1.94 |  | 6 | 230.67 | 21 | 21 |  | 42 | 2.53 | 0.303 | 212 |
|  | 13 |  | 120.0353 | 30.0721 |  | 1454 | 18 | 6 | 30 | 1.58 |  | 6 | 311.11 | 14 | 16 |  | 30 | 1.91 | 0.236 | 161 |
|  | 14 |  | 120.0349 | 30.0725 |  | 1454 | 18 | 7 | 42 | 1.48 |  | 6 | 224.22 | 6 | 3 |  | 9 | 0.89 | 0.124 | 124 |
|  | 15 |  | 120.0300 | 30.0371 |  | 1454 | 18 | 7 | 20 | 1.29 |  | 6 | 241.78 | 17 | 18 |  | 35 | 2.35 | 0.284 | 155 |
|  | 16 |  | 120.0256 | 30.0734 |  | 1454 | 18 | 9 | 24 | 1.34 |  | 6 | 317.33 | 13 | 10 |  | 23 | 2.24 | 0.291 | 181 |
|  | 17 |  | 120.0254 | 30.0737 |  | 1454 | 18 | 7 | 45 | 1.05 |  | 6 | 247.11 | 22 | 10 |  | 32 | 2.25 | 0.274 | 130 |
|  | 18 |  | 120.0252 | 30.0738 |  | 1454 | 18 | 6 | 30 | 1.25 |  | 6 | 222.22 | 19 | 8 |  | 27 | 2.05 | 0.256 | 145 |
|  | 19 |  | 120.0257 | 30.0728 |  | 1454 | 18 | 7 | 20 | 1.47 |  | 6 | 225.56 | 16 | 17 |  | 33 | 2.28 | 0.278 | 97 |
|  | 20 |  | 120.0305 | 30.0730 |  | 1454 | 18 | 7 | 25 | 1.59 |  | 6 | 200.00 | 13 | 13 |  | 21 | 1.75 | 0.229 | 174 |
|  |  |  |  |  |  |  |  |  |  |  |  |  |  |  |  |  |  |  |  |  |
| Qingshan Lake wetland | 1 |  | 119.4541 | 30.1532 |  | 1427 | 15.9 | 30 | 30 | 1.30 |  | 6 | 248.00 | 31 | 9 |  | 40 | 2.41 | 0.395 | 70 |
|  | 2 |  | 119.4538 | 30.1532 |  | 1427 | 15.9 | 26 | 30 | 1.23 |  | 6 | 200.00 | 5 | 6 |  | 11 | 0.59 | 0.082 | 80 |
|  | 3 |  | 119.4537 | 30.1530 |  | 1427 | 15.9 | 28 | 30 | 1.67 |  | 6 | 221.78 | 23 | 6 |  | 29 | 1.93 | 0.283 | 73 |
|  | 4 |  | 119.4537 | 30.1529 |  | 1427 | 15.9 | 29 | 15 | 1.50 |  | 6 | 220.67 | 38 | 10 |  | 48 | 2.65 | 0.476 | 50 |
|  | 5 |  | 119.4535 | 30.1527 |  | 1427 | 15.9 | 32 | 30 | 1.05 |  | 6 | 263.56 | 28 | 5 |  | 33 | 2.12 | 0.324 | 70 |
|  | 6 |  | 119.4534 | 30.1519 |  | 1427 | 15.9 | 25 | 35 | 1.10 |  | 6 | 209.56 | 0 | 1 |  | 1 | 0.00 | 0.000 | 36 |
|  | 7 |  | 119.4537 | 30.1515 |  | 1427 | 15.9 | 29 | 35 | 1.08 |  | 6 | 231.33 | 0 | 5 |  | 5 | 0.30 | 0.039 | 43 |
|  | 8 |  | 119.4538 | 30.1513 |  | 1427 | 15.9 | 37 | 35 | 1.07 |  | 6 | 219.33 | 11 | 3 |  | 14 | 1.00 | 0.131 | 43 |
|  | 9 |  | 119.4541 | 30.1512 |  | 1427 | 15.9 | 39 | 25 | 1.15 |  | 6 | 220.67 | 18 | 6 |  | 24 | 1.65 | 0.232 | 175 |
|  | 10 |  | 119.4544 | 30.1510 |  | 1427 | 15.9 | 41 | 35 | 1.16 |  | 6 | 220.00 | 13 | 7 |  | 20 | 1.41 | 0.192 | 96 |
|  | 11 |  | 119.4540 | 30.1529 |  | 1427 | 15.9 | 28 | 30 | 1.22 |  | 6 | 226.67 | 17 | 11 |  | 28 | 1.88 | 0.273 | 79 |
|  | 12 |  | 119.4544 | 30.1532 |  | 1427 | 15.9 | 31 | 32 | 1.79 |  | 6 | 240.00 | 32 | 8 |  | 40 | 2.77 | 0.310 | 65 |
|  | 13 |  | 119.4545 | 30.1534 |  | 1427 | 15.9 | 31 | 35 | 1.59 |  | 6 | 220.44 | 26 | 7 |  | 33 | 2.12 | 0.324 | 90 |
|  | 14 |  | 119.4548 | 30.1536 |  | 1427 | 15.9 | 32 | 38 | 1.53 |  | 6 | 225.78 | 20 | 4 |  | 24 | 1.65 | 0.232 | 53 |
|  | 15 |  | 119.4605 | 30.1427 |  | 1427 | 15.9 | 27 | 50 | 1.20 |  | 6 | 200.00 | 35 | 8 |  | 43 | 2.51 | 0.426 | 42 |
|  | 16 |  | 119.4601 | 30.1430 |  | 1427 | 15.9 | 32 | 45 | 1.14 |  | 6 | 221.33 | 45 | 20 |  | 65 | 3.00 | 0.350 | 70 |
|  | 17 |  | 119.4559 | 30.1433 |  | 1427 | 15.9 | 29 | 31 | 1.32 |  | 6 | 232.00 | 0 | 1 |  | 1 | 0.00 | 0.000 | 80 |
|  | 18 |  | 119.4609 | 30.1421 |  | 1427 | 15.9 | 26 | 42 | 1.30 |  | 6 | 208.00 | 20 | 13 |  | 33 | 2.12 | 0.324 | 73 |
|  | 19 |  | 119.4612 | 30.1420 |  | 1427 | 15.9 | 30 | 22 | 1.20 |  | 6 | 200.00 | 18 | 6 |  | 24 | 1.65 | 0.232 | 50 |
|  | 20 |  | 119.4606 | 30.1417 |  | 1427 | 15.9 | 31 | 30 | 1.49 |  | 6 | 216.67 | 35 | 12 |  | 47 | 2.44 | 0.277 | 70 |
|  | 21 |  | 119.4602 | 30.1414 |  | 1427 | 15.9 | 32 | 25 | 1.10 |  | 6 | 216.67 | 27 | 4 |  | 31 | 2.03 | 0.304 | 36 |

Notes: Long: Longitude(°), Lat: Latitude(°), Pre: Precipitation (mm), AAT: Annual average temperature (℃), Alt: Altitude (m), WT: Water transparency (cm), WSI: Water shape index, HA: Habitate age (years), CILUD: Comprehensive index of land use degree, PSP: Preservation of spontaneous plants(species), ICP: Introduction of cultivated plants (species), D: Patrick index, H: Shannon-Wiener index, J: Pielou index, TC: Total coverage (%)
